# Early changes in brain structure, functional connectivity and neuropsychiatric symptoms after HCV infection cure with direct-acting antivirals

**DOI:** 10.1192/j.eurpsy.2021.681

**Published:** 2021-08-13

**Authors:** M. Cavero, Z. Mariño, R. Navines, J. Pariente, E. Muñoz Moreno, C. Bartres, L. Nacar, S. Lens, S. Rodriguez-Tajes, S. Cañizares, N. Bargallo, X. Forns, R. Martin-Santos

**Affiliations:** 1 Psychiatry And Psychology Department, Centro De Investigación Biomédica En Red En Salud Mental (cibersam), Institut D’investigacions Biomèdiques August Pi I Sunyer (idibaps), Universitat Barcelona (ub), Hospital Clinic, Barcelona, Spain; 2 Liver Unit, Hospital Clinic Barcelona, CIBERehd, Institut d’Investigació Biomédica August Pi i Sunyer (IDIBAPS), Universitat Barcelona(UB), Barcelona, Spain; 3 Magnetic Resonance Image Core Facility, Institut d’Investigacions Biomèdiques August Pi i Sunyer (IDIBAPS), Barcelona, Spain

**Keywords:** Hepatitis C virus (HCV), functional connectivity, Neuropsychiatric symptoms

## Abstract

**Introduction:**

Hepatitis C virus (HCV) infection is known to be associated with neuropsychiatric manifestations as part of the disease. Previous neuroimaging studies showed brain connectivity dysfunction among HCV-infected patients

**Objectives:**

To assess, by MR in resting state, the potential structural and connectivity changes before (BL) and after HCV eradication (FU12) with direct-acting antivirals (DAA), along with clinical parameters.

**Methods:**

Twenty-one HCV-patients, aged≤55 years, without psychiatric history, nor advanced liver disease, and eligible for DAA, and 25 healthy controls were included. Evaluations were performed at BL and FU12. Brain volume and local gyrification index (LGI) were assessed in MR-T1, and functional connectivity by seed-based analysis (left insula). Depression (MADRS/PHQ9) and neurotoxicity symptomatology (NRS) were assessed. We compared patients between BL/FU12, and controls by means of paired/independent T-test analysis.

**Results:**

Substained virologycal response was obtained in all patients (100%). Depressive and neurotoxicity symptomatology improved after cure (p<0.01). HCV-patients showed a reduced volume in a right latero-occipital area compared to controls (CWP<0.005) in both BL and FU12. This difference was smaller between FU12 and controls. LGI was higher in FU12-HCV compared to BL-HCV. fMRI connectivity showed a high association between insula and occipital/parietal territories in patients than controls, being higher among BL-HCV and controls. Differences were limited to occipital areas among FU12-HCV and controls.

**Conclusions:**

Neuropsychiatric symptomatology improved after cure. Left insula is altered among HCV-patients in structured and connectivity (mainly occipital areas). After cure differences with controls were reduced, suggesting a partial restoration of brain connectivity.

